# Long-Term Changes of Physiological Reactions in Young Lipizzan Stallions During Exercise Testing

**DOI:** 10.3390/ani15172479

**Published:** 2025-08-23

**Authors:** Nina Čebulj-Kadunc, Robert Frangež, Peter Kruljc

**Affiliations:** 1 Institute of Preclinical Sciences, Veterinary Faculty, University of Ljubljana, Gerbičeva 60, 1000 Ljubljana, Slovenia; nina.cebulj.kadunc@vf.uni-lj.si (N.Č.-K.); robert.frangez@vf.uni-lj.si (R.F.); 2 Clinic for Breeding and Health Care of Horses, Veterinary Faculty, University of Ljubljana, Gerbičeva 60, 1000 Ljubljana, Slovenia

**Keywords:** Lipizzan breed, stallions, exercise testing, infrared thermography, physiological values

## Abstract

The aim of the study was to determine the fluctuations of selected physiological parameters in young Lipizzan stallions (*n* = 10) during the initial phase of their training as indicators of adaptation to a graded exercise load and stress exposure. For this purpose, four exercise tests (ExT) with lunging were carried out over a period of one year. Physiological parameters (gait speed, heart and respiratory rate (HR and RR), rectal and body surface temperature (RT and BST), and cortisol and lactate concentration (CORT and LAC)) were measured before and after training. In all ExT, gait speeds increased (*p* < 0.001) during the transitions from walk to trot and canter, followed by a significant (*p* < 0.001) increase in HR, RT, BST, and CORT, but not LAC values. However, the gate speed has no influence on the measured parameters. The highest BST values and corresponding warming were measured in the cranial region, followed by the caudal and distal body regions. The values of the measured variables remained within the ranges for warm-blooded horses, indicating adequate adaptation of the stallions to the applied stress level, but their variations could depend on air temperature or humidity. The results presented contribute to the knowledge of the complex physiological processes that occur in horses during exercise and point to the importance of environmental factors for adaptation to exercise.

## Simple Summary

Ten young Lipizzan stallions were tested four times over the course of a year with lunging exercises. The researchers checked the speed, heart rate, respiratory rate, rectal temperature, and skin temperature before and after exercise. They also measured cortisol and lactate concentrations. During the tests, the horses’ speed, heart rate, and temperature increased when they moved from a walk to a trot to a canter. Cortisol concentrations also increased, while lactate concentrations hardly changed. The highest skin temperatures were recorded on the front of the horses, the lowest on the cannons. The physiological values of the horses remained within the normal range, showing that they were well adapted to the exercise. These values were affected by the environment: speed, respiratory rate, and skin temperature increased at higher air temperatures and lower humidity, while heart rate showed an opposite trend. These results help us to understand how horses’ bodies respond to exercise and what role environmental factors play in their adaptation.

## 1. Introduction

Sport horses are often exposed to physical and emotional stress, leading to various diseases and injuries that affect their physical fitness and work performance [1,2]. In recent decades, exercise testing has proven to be a valuable tool that can help veterinarians and trainers to compare horses and assess their fitness level, performance potential, and training progress. Sport horses that are pushed beyond their limits in competition are often subject to sports injuries and stress. For this reason, the correct assessment of the physical performance and training responses of sport horses is of great importance for their welfare [1,2,3,4]. Through the simultaneous assessment of multiple body systems, including the respiratory, musculoskeletal, and cardiovascular systems, supported by laboratory testing, exercise testing has proven successful in identifying clinical signs that affect athletic performance, are undetectable at rest, and are often multifactorial [1,2,3,4].

Various standardized exercise tests (SET) have been described in horses and developed based on their physiological responses and adaptation to the workload, such as treadmill or field tests [1,2,3,5]. However, equine field tests have advantages over the use of a treadmill [2] and have been shown to be a more appropriate approach as they allow assessment under competition-like conditions and provide objective data on physiological parameters such as heart rate, speed, and blood lactate concentration [6,7].

The performance of horses during exercise testing depends on disturbances in thermoregulation, especially in hot and humid environments [8,9] or in horses that are not acclimatized to the ambient temperature [9,10]. Therefore, skin temperature can serve as a useful indicator of acute thermoregulatory fluctuations, which can be effectively detected by infrared thermography (IRT) [11,12,13,14,15,16,17,18]. IRT has been used in veterinary medicine since the 1970s and complements conventional X-ray and ultrasound examinations [14,19,20]. Recent advances in equipment design and imaging technology have expanded the use of this device in the diagnosis, prognosis, and evaluation of a variety of pathologic conditions, including those related to musculoskeletal, inflammatory, and neurologic diseases [17,20,21,22]. Thermography has also been shown to be useful in assessing equine welfare and acute stress levels [23,24]. It has also been used to assess the performance of horses in various activities such as exercise, training, and competition [11,15,16,20,25]. A study on thoroughbreds in racing [15] showed a correlation between body surface temperature and blood lactate concentration, highlighting the potential of IRT in assessing adaptation to increased workload during race training.

While advances in the study of equine exercise physiology have led to the development of scientifically based programs to improve the physical fitness of equine athletes [26,27], there are few scientific studies investigating the physical responses of young horses during their initial equestrian training, despite their known susceptibility to overtraining [26,28]. Young domestic horses are often raised in herds that provide them with ample opportunity for social interaction and unrestricted exercise. However, when the first riding training begins, these horses are often isolated from their herd and kept alone in boxes [29]. On the other hand, they are also exposed to a range of unfamiliar stressors, both anthropogenic, environmental, and social. All of these can affect their ability to cope with difficult situations, alter their behavior and physiological responses, and ultimately affect their welfare. Unfavorable conditions can impair the horse’s learning and training abilities, potentially reducing the value of the horse [30,31]. Inappropriate training methods or inadequate management can also lead to behavioral conflicts that pose a risk to the safety of riders and handlers [32]. Therefore, it is necessary to minimize the horse’s fear response to the trainer and to accustom the animal to unfamiliar situations and tasks that occur in the early stages of training [31,33].

**Figure 1:**
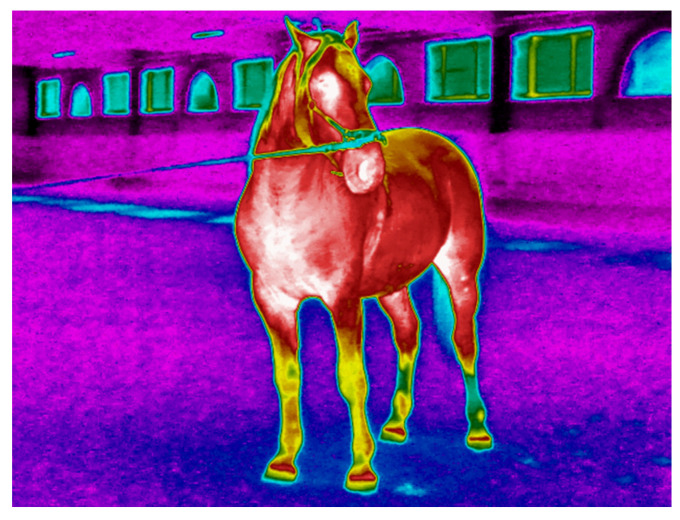
Thermogram of a young Lipizzan stallion before the start of the exercise test with different thermal patterns in the different body regions (warmest regions in white (31.9–33.7 °C) or red (26.2–30.7 °C) with an intermediate temperature in yellow (21–21.6 °C) and green (14.9–20.3 °C), and the coldest regions in blue (12.1–12.3 °C) and black color.

There is also limited data on appropriate training or exercise testing protocols tailored to specific equestrian disciplines such as dressage as opposed to show jumping, horse racing, or endurance riding [1,2,3,4,5,34]. Furthermore, it has been observed that different breeds of horses and even horses of different sexes show different physiological or biochemical responses to identical forms of exercise [2,28,35]. In our previous studies, which formed the basis for further research in the field of exercise testing and sports physiology of the Lipizzan horse breed, we reported the cardiovascular, respiratory, and thermoregulatory responses to incremental exercise in adult Lipizzans during lunging [36] and riding [37] and in Lipizzan fillies during lunging [16]. The Lipizzan horse breed, one of the oldest in Europe, is considered endangered due to its limited population and the scattered distribution of breeders [38]. Lipizzan stallions (Figure 1) are known for their exceptional performance in classical dressage, but the breed has also found favor in modern equestrian sports such as dressage and combined driving [38]. However, our knowledge of the physiological responses of Lipizzans to workload is limited, and there are few benchmarks for assessing their fitness during training or performance [16,36,37].

Therefore, the aim of the present study was to determine the baseline and post-exercise values of selected physiological (cardiovascular, respiratory, thermoregulatory, and blood biochemical) parameters in Lipizzan stallions during the initial training phase and to follow their changes as indicators of adaptation to a graded exercise load repeated over a year using standardized exercise tests. We hypothesized a significant increase in values of the investigated parameters after the exercise, but the difference between the values before and after exercise will decrease with time (training progression). By monitoring fluctuations in serum cortisol concentrations (CORT), we also aimed to assess the level of stress to which young stallions are exposed during the initial phase of their training. We expected a decrease in basal cortisol concentrations with progression of the study and a significant increase in these values after exercise in each test performed.

The results presented complement our previous findings from exercise testing in adult Lippizans [36,37] and Lipizzan fillies [16] and provide additional insights into the exercise physiology of Lipizzans that will contribute to the development of standards and protocols for monitoring fitness levels and training progress in this breed. Our results will also contribute to the general knowledge in the field of equine sports medicine and exercise testing, and will also be helpful in monitoring the health status and assessing the well-being of horses in general.

This manuscript is part of a comprehensive study of Lipizzan horses of various ages and stages of use that has been ongoing for several years, investigating physiological characteristics and athletic qualities, as well as resistance to various forms of stress, are being investigated with the aim of developing a standardised stress test for this breed.

## 2. Materials and Methods

### 2.1. Animals

The study took place at the Lipizza Stud Farm in Slovenia (Lipica Stud Farm, Lipica 15, Sežana), the oldest European stud farm dedicated exclusively to the fundamental activity of breeding Lipizzan horses, one of the most ancient, domesticated horse breeds [38]. Ten purebred Lipizzan stallions born in 2018 and four years old at the time of the first exercise test took part in the study. Their initial average body weight was 457 ± 6.4 kg, and reached 475 ± 21.2 kg at the end of the study. They were housed in a stable designed for stallions in individual boxes (10 m^2^) filled with sawdust and equipped with an automatic water supply to ensure unlimited access to drinking water. Each horse received 10 kg of hay, 4 kg of pressed oats, and 0.1 kg of the vitamin and mineral mixture Kovisal (Jata Emona, d.o.o., Agrokombinatska 84, Ljubljana, Slovenia) daily.

The stallions were selected by the stud’s experts among the individuals that satisfied the breeding goals of the Lipizzan breed, considering their genotypical, phenotypical and psychological characteristics for training of classical dressage, including *haute école*, and successful passed the veterinary examination. Based on the individual medical history and clinical examinations, which included the detection of lameness and continuous monitoring of physiological parameters, including haematology and serum biochemistry, the stallions examined were considered clinically healthy throughout the study. The stallions were handled with the traditional procedures of the stud farm, in accordance with the professional and ethical equestrian standards in line with the official Slovenian standardised breeding programme for Lipizzans [39], which was detailed in our previous article [16]. The procedures used in the present study were carried out in accordance with the protocol approved by the Animal Welfare Commission of the Veterinary Faculty, University of Ljubljana, Gerbičeva 60, Ljubljana, under permit No. 38/13. This provides transparency and alignment with ethical standards in animal research.

### 2.2. Test Protocol and Physical Activity

The study consisted of four consecutive exercise tests (ExT) with identical protocols. The first exercise test (ExT-1) was performed in May 2022, followed by the next tests in September 2022 (ExT-2), January 2023 (ExT-3), and June 2023 (ExT-4), each between 8 and 11 a.m. Each ExT was conducted by lunging in an indoor arena (20 m × 50 m) with a sand floor that allowed a lunging radius of 8.5 m. The leading and lunging of each stallion was carried out by a professional trainer who was responsible for the stallion throughout the entire introduction process. Each ExT consisted of 3 phases with specific activities (before exercise (BEx), during exercise (Ex), and after exercise (AEx). The reins were changed at each gait passage. The protocol of the exercise tests was described in detail in our previous study [16] and is presented in Table 1 [16].

### 2.3. Equipment, Measurements, and Sampling

This experiment follows the research methods previously used to measure heart rate, rectal temperature (RT), and respiratory rate (RR), body surface temperature (BST), and blood samples for the determination of serum and salivary cortisol and plasma lactate in horses. For further details, please refer to reference [16].

### 2.4. Laboratory Analyses

The laboratory analyses of serum, salivary cortisol, and plasma-lactate follow the methods already used in our experiment. Further details can be found in reference [16].

### 2.5. Data Analyses

Statistical analyses were performed with the same methods used in our experiments. For further details, please refer to reference [16]. We also used an analysis of covariance (ANCOVA) to identify variance changes in the dependent variables caused by the covariate (speed), and separate these from the variance due to qualitative factors. The data were analyzed with SigmaPlot 14.5 (Systat Software, Inc., Erkrath, Germany). Data were first tested for normality (Shapiro–Wilk test) and homogeneity of variance (Levene’s test) to assign them to parametric or non-parametric analyses. Subsequently, an analysis of equal slopes model was used, followed by all pairwise multiple comparison procedures (Holm–Sidak method) to evaluate whether different movement speeds in the individual ExTs affected the values of dependent variables.

## 3. Results

### 3.1. Gait Speeds During Exercise

In all exercise tests, a significant increase in gait speed (*p* < 0.001) was observed in the transitions from walk to trot to canter (Table 2). The highest speeds in walk, trot, and canter were measured in ExT-4 and were higher (*p* < 0.001) than those measured in ExT-1, 2, and 3, which were not different from each other (*p* > 0.05). A positive correlation was observed between the speeds in walk and trot (R = 0.980, *p* < 0.05), walk and canter (R = 0.980, *p* < 0.05), and trot and canter (R = 1, *p* < 0.001). The gait speeds correlated negatively with humidity (walk: R = −0.948, trot: R = −0.976, canter: R = −0.970; *p* < 0.05, respectively) and positively with air temperature (R = 0.620 to 0.639; *p* > 0.05, respectively).

### 3.2. Heart Rate (HR)

**Figure 2:**
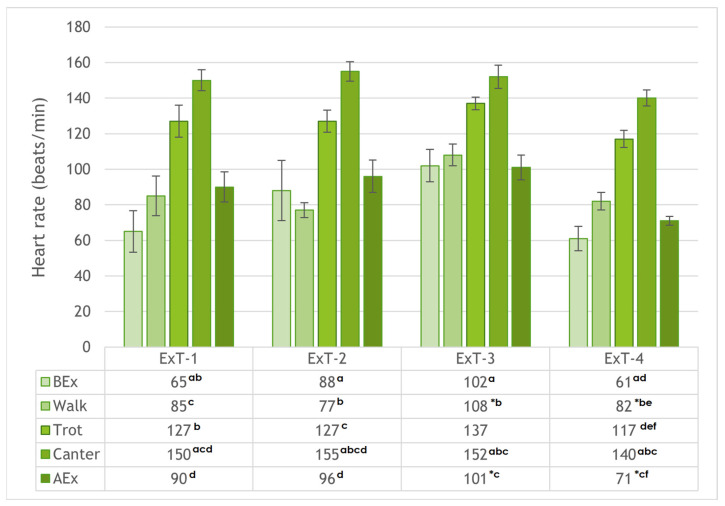
Heart rates (mean ± SE) before exercise (BEx), at walk, trot, and canter, and after exercise (AEx) in exercise tests 1 to 4 (ExT-1 to ExT-4). ^a–f^ *p* < 0.001 (for values in a column with the same character); * *p* < 0.05 (for values in a row).

Heart rate (Figure 2) increased progressively (*p* < 0.001 between phases) during the transition from resting BEx to walk and from trot to canter in all Ex tests. Subsequently, heart rate decreased in all Ex tests at rest AEx (*p* < 0.001 compared to canter). When comparing horses within specific phases of the Ex test, HR values were higher in ExT-3 than in ExT-4 at walk and at rest AEx (*p* < 0.05). There were no differences (*p* > 0.05) in HR values between Ex tests 1 to 4 at rest BEx, trot, and canter. The HR values correlated negatively with air temperature (from R = −0.938 for trot to R = −0.572 for canter; *p* > 0.05) and positively with humidity (from R = −0.943 for trot to R = 0.622 for walk; *p* > 0.05).

### 3.3. Rectal Temperature and Respiratory Rate

The mean rectal temperatures (RT) and respiratory rates (RR) of the investigated stallions examined in all four Ex tests, Bex, and AEx are shown in Table 3. Before Ex, the lowest RT was measured in ExT-3 (*p* < 0.01 compared to ExT-2, with the highest measured value). After Ex, the highest RT was measured in ExT-1 (*p* < 0.001 compared to all other Ex tests). RTs increased after Ex in all tests (see Table 3 for significance). Before Ex, RT correlated negatively with humidity (R = −0.502, *p* > 0.05) and correlated positively with ambient temperature (R = 0.917, *p* > 0.05), while in AEx there was only a slightly negative and insignificant correlation (*p* > 0.05) of RT with humidity (R = −0.401) and ambient temperature (R = 0.149) (Table 4).

The highest RR values BEx were measured at ExT-1 and the lowest at ExT-3 (*p* > 0.05). After Ex (Table 3), they increased (*p* < 0.001 for ExT-1 and ExT-4, *p* < 0.01 for ExT-2), reaching the highest values in ExT-1 and the lowest in ExT-3 (*p* < 0.001). A significant positive correlation (R = 0.961; *p* < 0.05) was found between the RRs before and after Ex. RR correlated positively with humidity (R = 0.544 and R = 0.753, *p* > 0.05 BEx or AEx), but slightly negative with ambient temperature (R = −0.268 and R = −0.445, *p* > 0.05 BEx or AEx).

### 3.4. Body Surface Temperatures

**Figure 3:**
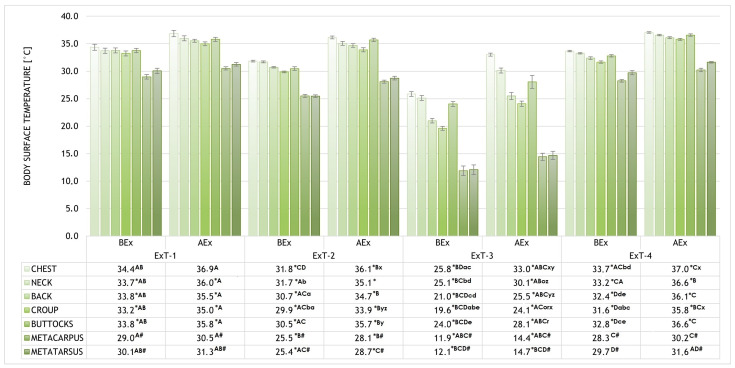
The body surface temperatures (BST) of selected regions (chest, neck, back, croup, buttocks, metacarpus, and metatarsus) before and after exercise (BEx and AEx). * *p* < 0.001 (for temperatures of the specific region within an ExT test BEx vs. AEx); ^A–D^ *p* < 0.001 (for temperatures of the specific region within the phases (BEx or AEx) of various exercise tests with the same label); ^#^ *p* < 0.001 (for metacarpus and metatarsus compared to all other regions BEx and AEx in ExT-1 and Ext-2; ^a–e^ *p* < 0.001 (for temperatures of different regions BEx within an Ex-T with the same label); ^o,r,x–z^ *p* < 0.001 (for temperatures of different regions AEx within an Ex-T with the same label).

The body surface temperatures (BST) of selected regions (chest, neck, back, croup, buttocks, metacarpus, and metatarsus) before and after exercise (BEx and AEx) are shown in Figure 3 for all four exercise tests performed (ExT-1 to 4). The statistical significance of the results is also included alongside the graphical representation of the results. Before Ex, the highest BSTs for all regions were measured in ExT-1, the lowest in ExT-3, and intermediate in ExT-2 and ExT-3 (*p* < 0.001; see Figure 3 for details). Within each test, the highest BSTs were measured for the chest and the lowest for the metacarpus and metatarsus (*p* < 0.001) before Ex. Intermediate BSTs were measured for neck, back, croup, and buttocks (*p* > 0.05). After the Ex test, the BSTs of almost all regions increased in all Ex tests (*p* < 0.001), except for the breast in ExT-1 (*p* < 0.05), and metacarpus and metatarsus in ExT-1 and Ext-4 (*p* > 0.05). BSTs correlated positively with air temperatures both before and after Ex. In contrast, a negative correlation was found between the BSTs and humidity before and after the Ex test, but this was not significant. The correlation coefficients and the statistical significance of the results are shown in Table 5.

**Figure 4:**
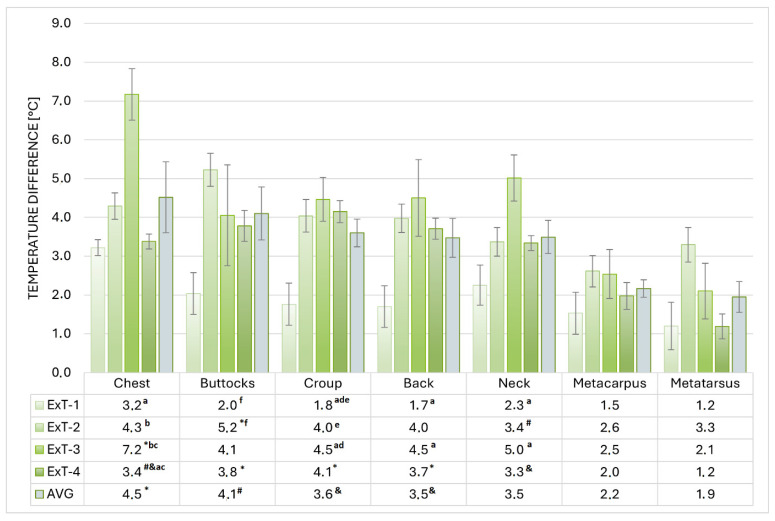
Warming of selected regions in exercise tests 1 to 4 (ExT-1 to ExT-4). ^a–c^ *p* < 0.001; ^d,e^ *p* < 0.01; ^f^ *p* < 0.05 (for values within a column with the same label); * *p* < 0.001; ^#^ *p* < 0.01; ^&^ *p* < 0.05 (for marked values versus metacarpus and metatarsus within a row).

**Figure 5:**
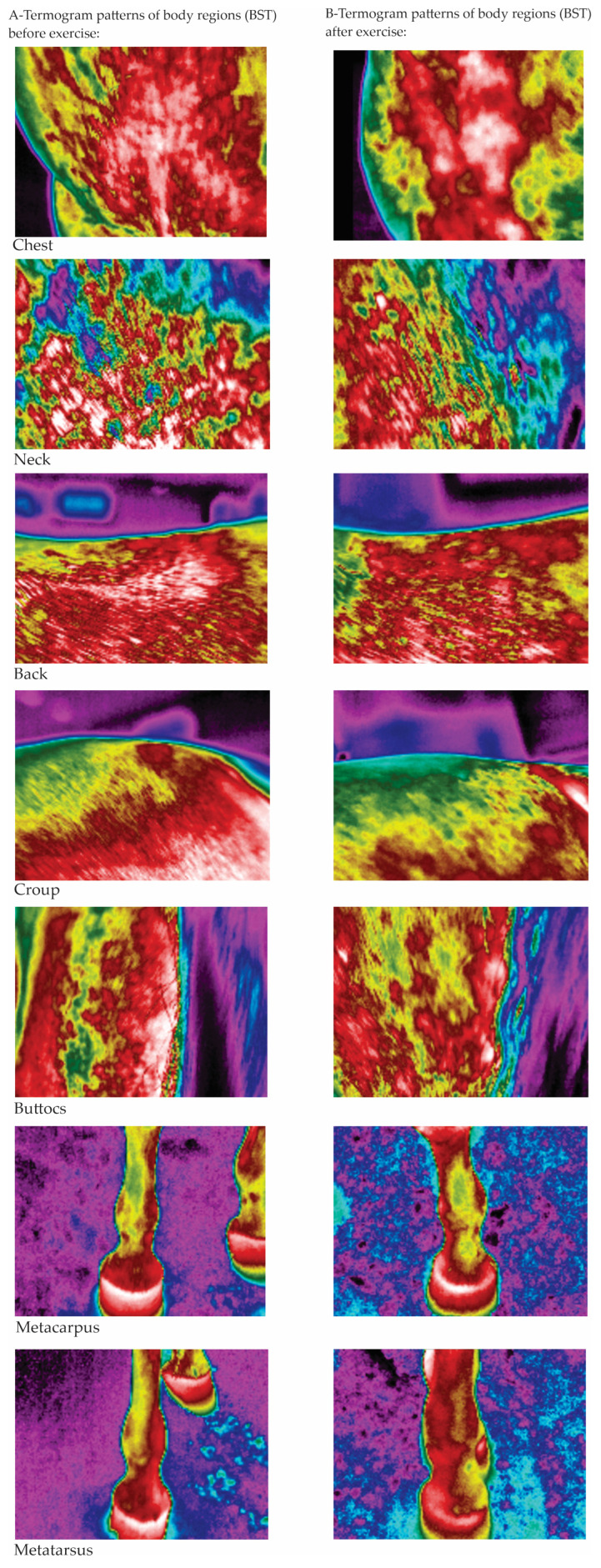
The thermographic patterns of BST before (**A**) and after (**B**) exercise test (warmest regions in white (31.9–33.7 °C) or red (26.2–30.7 °C) with an intermediate temperature in yellow (21–21.6 °C) and green (14.9–20.3 °C), and the coldest regions in blue (12.1–12.3 °C) and black color).

The highest average warming (difference between the BST before and after Ex) was measured in ExT-3 (4.3 ± 0.3 °C) and the lowest (2.0 ± 0.2 °C) in ExT-1 (*p* < 0.001), which was also lower than in ExT-2 and ExT-3 (3.8 ± 0.2 °C and 3.2 ± 0.1 °C, respectively). The regions that warmed up the most on average were the chest and buttocks, followed by the croup, back, and neck, while the metacarpus and metatarsus warmed up the least. Within each ExT, a similar distribution of warming was observed, with the highest values in ExT-3 and the lowest in ExT-1 (for absolute values and statistical significance, see Figure 4). Positive correlations (R = 0.954 for croup BEx to R = 0.993 for neck AEx) were observed between the BSTs and air temperatures, which were significant (*p* < 0.05) for all investigated regions. Correlations between BSTs of investigated regions and air humidity were negative (R = −0.572 for croup to R = −0.714 for neck) and insignificant (*p* > 0.05); see details. The thermographic patterns of BST before and after exercise tests can be seen in Figure 5.

### 3.5. Biochemistry

The measured CORT concentrations in serum and saliva (Table 6) remained similar in all 4 Ex tests (*p* > 0.05) and increased AEx (*p* < 0.001 for serum, *p* < 0.01 for saliva in ExT-1) with the exception of salivary CORT, which decreased AEx in ExT-2 (Table 6). CORT values in saliva and serum correlated positively (R = 0.461; *p* < 0.001). No significant differences between tests were observed in LAC concentration, which varied between 0.35 ± 0.07 nmol/L and 0.44 ± 0.06 nmol/L before Ex (*p* < 0.05) and increased (*p* > 0.05) and reached values between 0.62 ± 0.2 nmol/L and 1.0 ± 0.3 nmol/L after Ex (Table 6).

## 4. Discussion

In the present study, conducted on young Lipizzan stallions, the response of physiological variables to a graded workload was assessed by measurements before and after exercise in four exercise tests performed over one year. This allowed us to observe the long-term adaptation of the young stallions to the workload and stress induced by the different procedures during the initial training period, but also to capture the different environmental conditions manifested by seasonal variations in ambient temperature and humidity. The study is a continuation of our research in the field of exercise testing in adult Lippizan horses [36,37] and Lipizzan fillies [16].

### 4.1. Gait Speeds

The gait speeds of the Lipizzan stallions studied were in the ranges previously reported for riding horses [5,40,41], but faster than in our previous studies conducted with adult Lipizzans [36] and Lipizzan fillies [16] during lunging. These results suggest that the gait speeds of the Lipizzan stallions studied are independent of the movement on the circle during lunging, as previously observed in adult Lipizzans and fillies [16,36], where the slower movement was attributed to the use of a less efficient combination of stride frequency and stride length during lunging on the circle compared to movement on the straight, as also suggested in another study [5]. Another, more likely explanation for the Lipizzaner stallions’ faster gaits is the way they responded to the trainers’ commands. Indeed, as in our previous studies [16,36], almost any physical constraint to accelerate the pace was avoided, apart from the voice, changing the tension of the lunging rope, and pointing the whip at the horse’s hindquarters, which were obviously stimuli that elicited a more intense response in stallions than in fillies or geldings. When considering these differences in gait speeds, it must also be taken into account that the exercise tests in Lipizzan fillies covered a relatively short period of two months [16], whereas the tests in the present study were carried out over a year. During this time, the stallions were lunged daily according to the Lipica Stud Farm protocol and successfully acclimatized to running in circles, which allowed them to reach higher speeds. The adult Lipizzaners [36], on the other hand, were used for riding and were less accustomed to lunging, which was only performed during the exercise tests.

As already reported for horses [5,16,36,41], the gait speeds of the Lipizzan stallions examined increased in all exercise tests during the transitions from walk to trot to canter and were within the usual ranges for horses [5,41]. The speeds in all gaits recorded in Lipizzan stallions during ExT-4 were faster than in the other three exercise tests, which did not differ from each other. This indicates an improvement in movement quality and overall physical performance [27,33] for the successful completion of the work initiation of the Lipizzan stallions studied. Positive correlations were found between the speeds at walk, trot, and canter of the Lipizzan horses examined in all the Ex-tests carried out. This means that a horse that moves faster in walk also moves faster in trot and canter (and vice versa) and reflects the individual’s motor abilities or the influence of physiological factors that were not observed in this study, including stride length, which determines speed in each gait. Thus, horses with longer strides achieve higher maximum gait speeds than those with shorter strides [42], which may explain our results in the Lipizzan stallions. The effects of temperature and humidity, especially when they reach high levels, are known factors that influence the physical performance of horses [10,43]. In sub-Mediterranean climates, temperature and humidity rarely reach extreme values. Nevertheless, we were able to perform our measurements on Lipizzan stallions under different environmental conditions (Table 2) and found that gait speed correlated negatively with humidity and positively with air temperature. Our results demonstrate the importance of monitoring ambient temperature and humidity for the assessment of physical performance, even in temperate climates [10,43].

Gait speed was not standardized, as the tests were conducted in a closed indoor arena. Therefore, ANCOVA was applied to reduce unexplained variability (error), resulting in clearer outcomes and increased analytical power. The analysis showed that different movement speeds of young Lipizzaner stallions had no significant effect (*p* > 0.05) on the measured parameters (BST, RT, RR, HR, CORT, and LAC).

### 4.2. Heart Rate (HR), Respiratory Rate (RR), and Rectal Temperature (RT)

The heart rate (HR) of horses is determined by the intensity of exercise and is an indicator of their metabolic capacity. This response is influenced by various external and internal factors, such as environmental conditions, the horse’s fitness level, and its general state of health [1,28,35,44]. The mean resting HR of Lipizzan stallions was above the physiological range for warmbloods [5,16,36,45,46,47]. This suggests that their arousal was triggered by leaving the stalls, being equipped with heart rate monitors, and encountering unfamiliar people prior to the exercise test. A comparable psychogenic HR response has been observed in racehorses just before the start of a competition and in warmblood horses during handling, saddling, or harnessing prior to exercise [1,3,5,16,45,47].

During exercise, HR fluctuations in Lipizzan stallions followed a similar pattern in all exercise tests, with a significant progressive increase during the transition to faster gaits. Within ten minutes of the exercise, HR values decreased in all four tests compared to the canter, indicating a normal physiological response and a good fitness state of the tested stallions. The heart rates were within the ranges reported for other horse breeds in comparable studies and did not reach the maximum HR, which can exceed 200 beats/min in horses [5,8,16,36,40,44,45,46]. When comparing values in the same phases, there were no differences (*p* < 0.05) in heart rates between Ex tests 1 to 3. The only exception was Ex-T4 with lower heart rates in all phases (significant for walking and rest after Ex). We believe that this result reflects the influence of environmental factors [10,43] on the performance of the studied stallions, as HR values were negatively correlated with air temperature, which reached the highest value at ExT-4, and positively correlated with humidity, which reached the lowest value at ExT-4.

The mean rectal temperatures (RT) measured in the Lipizzan stallions were within a range previously reported in horses [12,46] and also measured in our previous studies on Lipizzans [16,36]. Before Ex, the lowest RT was measured in ExT-3 and the highest in ExT-2, and a positive but insignificant correlation with ambient temperature was found. RT values correlated positively with ambient temperature. In fact, the lowest RT in ExT-3 was measured in winter at an ambient temperature of 6.3 ± 0.5 °C, which was close to the lower critical temperature of horses [10,48,49,50,51]. On the other hand, the differences in resting RT were not different in the other three tests (ExT-1, 2, and 4) performed at ambient temperatures within the equine thermoneutral zone. After Ex, RTs increased in all Ex tests, reflecting the increased heat production during physical activity [13,16,36].

The highest RR values in the studied Lipizzan stallions BEx and AEx were measured in ExT-1, the lowest in ExT-3. Despite the differences between tests, all values remained within a normal range for warmbloods [46,47], which was also confirmed in our previous studies on Lipizzans [16,36]. After exercise, mean RR values increased in all Ex tests. These changes reflect the physiological responses to the increased oxygen consumption of working skeletal muscle during exercise [6,47]. In addition, increased RR also contributes to the evaporative loss of heat from the airways due to increased heat production during exercise [12]. In all Ex tests, a strong positive correlation was found between RR values before and after Ex, indicating an influence of RR values at rest on RR values during exercise. The measured RR values BEx and AEx correlated positively with humidity, indicating the role of humidity in the thermoregulation of the Lipizzan horses studied. Similar correlations were found in other studies in horses [8,12,13,46,47] and in Lipizzan fillies [16]. On the other hand, a small negative correlation between mean RR values measured before and after Ex was not strong enough to confirm a causal correlation of RR with ambient temperature [8,12,13,46,47].

### 4.3. Body Surface Temperature (BST)

Through their metabolic activity, resting muscles generate a constant amount of heat that increases in proportion to the intensity of the workload during exercise. To maintain body temperature within the physiological range, various thermoregulatory mechanisms are activated, including increased blood flow through the skin capillaries, resulting in an increase in body surface temperature [12,13,18,52]. Horses exhibit bilateral symmetry of the BST [11,12,16,18,36], a finding that was also confirmed in this study with Lipizzan stallions in all Ex-tests. This indicates balanced muscle work and a suitable track surface during exercise. As a result, the average BST values measured in the present study were calculated and further analyzed considering both sides of each inter-test region, as previously suggested [16,36]. In Lipizzan stallions at rest, the highest average BST values of all regions were measured in ExT-1, followed by ExT-2 and ExT-4, and the lowest in ExT-3. Within each test prior to Ex, the highest resting BSTs were measured for the chest and the lowest for the metacarpus and metatarsus. Mean BST values were measured for the neck, back, croup, and buttocks, while the coldest regions were the metacarpus and metatarsus. A similar distribution of BST was also found in our previous study in Lipizzan fillies [16]. The BST values measured in Lipizzan stallions in this study were within the ranges reported in other equine studies [11,12,18,47,52] and previously measured in adult Lipizzans [36]. The only exception was the ExT-4, where the average BSTs were similar to those measured in Lipizzan fillies [16]. Just like the Ex tests in Lipizzan fillies, the ExT-4 in this study was performed in winter at a similar air temperature of approximately 6 °C, indicating the influence of air temperature close to the lower critical temperature of horses on BSTs [10,48,49,50,51].

Numerous studies on horses have documented changes in the distribution of surface temperatures in different regions of the body before and after exercise, confirming the effects of exercise on skin heating [11,12,16,18,36,52,53,54,55]. The BST of the Lipizzan stallions examined increased in all tests after exercise. The chest showed the highest BST value in all four tests, followed by the neck and buttocks [12,54], suggesting that the greatest concentrations of maximum temperatures are located over the anatomically largest surfaces, which play an important role in thermoregulation. In contrast to Lipizzan fillies [16], where the highest temperature was measured at the neck, in this study, we measured the highest BST at the chest, indicating a different utilization of these regions and a different distribution of the center of gravity in stallions compared to fillies. The neck, back, croup, and buttocks showed a medium BST value, while the metacarpus and metatarsus showed the lowest heating [11,12,16,18,36,52,53,54,55]. Despite some significant differences within a single Ex test, the BSTs were evenly distributed and indicated a good balance of all body parts of the Lipizzan stallions studied during exercise. As the workload intensifies, the metabolic rate and oxygen demand also rise, prompting greater blood flow to the muscles. These reactions ultimately enhance blood flow to the skin and facilitate the release of excess heat into the environment [12,18,27,52]. The only exception was ExT-3, which was performed in winter. Here, we found greater variability in the distribution of BST and also lower values compared to other Ex tests conducted at higher air temperatures [10,48,49].

During our study, average ambient temperatures were consistent with those reported in previous studies [11,12,36,50,51,52,55]. The only exception was ExT-3, which was conducted in winter at an average temperature of 6.3 ± 0.5 °C. This could explain the lower BST values compared to the other 3 Ex tests and is similar to the BST values measured in Lipizzan fillies tested in winter [16]. The increased heat loss, which is proportional to the decrease in air temperature, is primarily due to reduced peripheral blood flow as blood is directed to the internal organs, as well as thermal insulation by skin, hair coat, and subcutaneous fat [47,56], and could also depend on the type of horse [56]. There is an almost linear inverse correlation between the gradual decrease in ambient temperature towards the lower critical temperature (5 °C) and the rate of non-evaporative heat loss in horses [10,48,49,50,51], which is confirmed by variations in limb surface temperatures that are directly related to changes in air temperature between 5 and 25 °C [47]. In this study, BSTs of all investigated regions in Lipizzan stallions correlated positively with ambient temperature and negatively with humidity. In our opinion, this is due to the inverse relationship between ambient temperature and humidity that we found in this study. Similar correlations have been found in other studies in horses [8,12,13,46,47,57] and in Lipizzan fillies [16].

In the Lipizzan, the highest average warming (the difference between the BST before and after Ex) was measured in ExT-3 and the lowest in ExT-1, which was also lower than in ExT-2 and ExT-3. The regions that warmed up the most on average were the chest and buttocks, followed by the croup, back, and neck, while the metacarpus and metatarsus warmed up the least. Within each ExT, a similar distribution of warming was observed, with the highest values in ExT-3 and the lowest in ExT-1. The temperature differences between pre- and post-exercise values for the different body regions of Lipizzan stallions were within the ranges reported for horses with comparable workloads [11,16,36], but higher than those observed in horses after jumping competitions [12] or felin ponies [52] exposed to light treadmill exercise. Our results show that the heating of the different body regions after Ex is independent of the ambient temperature and confirm the different contributions of the different regions to thermoregulation and the different loads they bear in supporting and moving the body [11,12,52,53,54,55].

### 4.4. Cortisol and Lactate Concentrations

Changes in serum CORT concentration are objective and useful indicators for the assessment of stress in horses [58]. A variety of situations to which horses are exposed, including physical training, riding competitions, and veterinary examinations, are classified as potential stressors [33], which was also the case in our study. Physical exercise is recognized as one of the most physiologically demanding stressors, leading to a temporary disruption of various homeostatic parameters, requiring key regulatory systems to work together to restore balance [58,59,60]. Physical exercise acts as a stimulus for increased cytokine production [61]. However, cortisol plays a crucial role in the feedback loop by suppressing the further release of cytokines during exercise, thus helping to restore homeostasis in the inflammatory response and helping to prevent the development of pathological conditions. In response to stress, signals from the brain trigger the production and release of hormones into the bloodstream, which are regulated by feedback mechanisms that either enhance or suppress secretion. This hormonal response is an adaptation mechanism that mobilises energy reserves and enables the organism to cope with new challenges. Specifically, acute stress stimulates the pituitary gland to release adrenocorticotropic hormone (ACTH), which in turn triggers the adrenal glands to secrete cortisol [62,63]. The measurement of cortisol concentrations in the blood is considered the gold standard for assessing stress, as its increase is closely linked to the intensity of acute stress [64]. Both physical and psychological stress have been proposed as tools to assess the body’s ability to regulate inflammation [65,66]. It is known that the glucocorticoids and catecholamines released during acute stress modulate the inflammatory response [67]. Under stress, the hormonal and immune systems interact to restore homeostasis [68,69]. If the organism is unable to restore homeostasis, this can have a negative impact on both the animal’s health and its athletic performance. The average CORT concentrations in the Lipizzan stallions in all four Ex-tests were in the lower range of the normal ranges reported for resting horses [3,10,41,70,71,72]. An increase in CORT in plasma or saliva is observed in horses after different types of exercise [10,33,71,72,73], which was also observed in our study in each Ex test, comparing BEx and AEx values in serum or saliva. This clearly shows a response of the hypothalamic-pituitary-adrenal axis in the studied Lipizzans to the increased metabolic demands during exercise [10]. Due to the design of our study, we were unable to distinguish between the responses of the horses studied to exercise or other potential stressors, such as contact with unfamiliar people or environments. Although the correlations between circulating and salivary CORT are well known and were also confirmed in the present study [29,33], it is not possible to compare the values measured in different sample types (serum vs. saliva). Nevertheless, salivary CORT concentrations achieved in response to exercise were lower than in horses during road transport [33].

Lactate is continuously produced in active muscles and remains at a low level until its production exceeds excretion. In this case, the excess lactate is excreted to allow other organs to recycle their energy resources via the Cori cycle [74,75]. In addition to clinical applications, lactate can also be measured during exercise to assess the fitness and performance of sport horses [15,28,75,76]. In this context, lactate accumulation serves as an indicator of fatigue following anaerobic exercise, suggesting that fatigue is mitigated by the breakdown of lactate [77]. The normal lactate concentration in the blood of a resting horse is 1–2 mmol/L [16,28,74,75,77]. Similar values have been observed in Lipizzan stallions at rest before Ex. With increasing speed, the lactate concentration in the blood of horses increases exponentially and reaches 25 to 30 mmol/L after maximum speeds or intensive physical exertion [40,76]. Contrary to our expectations, the lactate concentration in the Lipizzan stallions tested did not reach the anaerobic threshold of 4.00 mmol/L after Ex in any of the 4 Ex tests performed [76,77]; only in Ex tests 2 and 3 was a slight increase over the baseline value observed, which exceeded 1 mmol/L. These low lactate values indicate low lactate production and rapid lactate clearance in the studied stallions, which is probably due to the low intensity of the exercise tests or good physical condition, where most of the energy for muscle work is generated by aerobic metabolism [76].

## 5. Conclusions

The results of our study show the physiological responses of young Lipizzan stallions to graded exercise over a period of one year. The values of cardiovascular, thermoregulatory, hematologic, and biochemical parameters measured at rest before exercise were within normal values for horses and increased during exercise due to the increased metabolic demands. Furthermore, their changes after the start of the study indicate a gradual adaptation of the stallions to the work routines, a gradual improvement in physical condition, and an adaptation to the environmental changes. Our study also found correlations between the variables studied and ambient temperatures or humidity, which underlines the importance of recording these environmental factors and considering their influence on the measurement results. This study contributes to the knowledge of the complex physiological processes that occur during exercise and provides a basis for further research in the field of equine exercise testing, sports medicine, and equine welfare. The results presented can also be seen as an important contribution to the preservation and development of the Lipizzan horse breed.

## Figures and Tables

**Table 1:** Presentation of the activities and measured parameters at each phase of the exercise test (BST—body surface temperature, RT—rectal temperature, RR—respiratory rate, HR—heart rate, and VP—blood sampling) [16].

Phase of the Test	Duration of Activity [min]	Activity	Recordings and Sampling *
Before exercise (BEx)	10	Rest	BST, RT, RR, HR, VP **
During exercise (Ex)	5	Lunging (walk)	HR
	5	Lunging (trot)	HR
	5	Lunging (canter)	HR
After exercise (AEx)	10	Rest	BST, RT, RR, HR, VP **

* air temperature and pressure were measured throughout the testing; ** for lactate and cortisol determination.

**Table 2:** Gait speeds (mean ± SE) in walk, trot, and canter during exercise in tests 1 to 4 (ExT-1 to ExT-4).

Variable	ExT-1	ExT-2	ExT-3	ExT-4
Walk (km/h)	7.5 ± 0.8 ^a^	8.0 ± 0.5 ^a,b^	7.2 ± 0.8 ^a,b^	15.4 ± 0.8 ^a,b,^*
Trot (km/h)	11.9 ± 1.0	16.1 ± 0.5 ^b^	12.1 ± 1.0 ^b^	26.9 ± 1.4 ^b,c,^*
Canter (km/h)	16.0 ± 1.2 ^a^	22.0 ± 0.7 ^a^	16.8 ± 1.4 ^a^	37.3 ± 3.3 ^a,c,^*

^a–c^ *p* < 0.001 for values in a column with the same character; * *p* < 0.001 for ExT-4 compared to ExT-1, 2 and 3.

**Table 3:** Rectal temperature (RT; mean ± SE) and respiratory rate (RR; mean ± SE) in Lipizzan stallions before and after exercise (BEx and AEx) in exercise tests 1 to 4 (ExT-1 to ExT-4).

Variable	Phase of the Test	ExT-1	ExT-2	ExT-3	ExT-4
RT [°C]	BEx	37.6 ± 0.05 ^a^	37.35 ± 0.05 ^c,B^	36.96 ± 0.3 ^a,B^	37.47 ± 0.06
	AEx	39.17 ± 0.16 ^a,^*	37.99 ± 0.22 ^c^	37.81 ± 0.14 ^a^	38.03 ± 0.14
RR [/min]	BEx	22.6 ± 2.6 ^a^	16.0 ± 1.7 ^b^	17.1 ± 2.7	20.8 ± 1.7 ^a^
	AEx	66.6 ± 8.2 ^a,D,B,A^	41.9 ± 3.8 ^b,B^	37.6 ± 4.4 ^A,C^	61.1 ± 4.9 ^a,D,C^

^a^ *p* < 0.001; ^b^ *p* < 0.01; ^c^ *p* < 0.05 for values in a column with the same label; ^A^ *p* < 0.001; ^B,C^ *p* < 0.01; ^D^ *p* < 0.05 for values in a row with the same label; * value is higher (*p* < 0.001) than other values in a row.

**Table 4:** Mean air temperature and humidity during exercise tests 1 to 4 (ExT-1 to ExT-4).

Variable	ExT-1 (May 2022)	ExT-2 (September 2022)	ExT-3 (January 2023)	ExT-4 (June 2023)
Temperature [°C]	20.6 ± 0.1	19.2 ± 0.2	6.3 ± 0.5	24.4 ± 0.5
Humidity [%]	71.4 ± 2.6	65.5 ± 2.9	77.0 ± 3.2	49.6 ± 3.9

**Table 5:** Correlation between the BST of selected body regions before and after exercise with air temperature and humidity (R–correlation coefficient; *p*–statistical significance).

Body Region	Air Temperature				Air Humidity			
	Before Exercise		After Exercise		Before Exercise		After Exercise	
	R	*p*	R	*p*	R	*p*	R	*p*
Chest	0.961	*p* < 0.05	0.988	*p* < 0.05	−0.601	*p* > 0.05	−0.686	*p* > 0.05
Neck	0.969	*p* < 0.05	0.993	*p* < 0.01	−0.617	*p* > 0.05	−0.714	*p* > 0.05
Back	0.957	*p* < 0.05	0.984	*p* < 0.05	−0.578	*p* > 0.05	−0.681	*p* > 0.05
Croup	0.954	*p* < 0.05	0.988	*p* < 0.05	−0.572	*p* > 0.05	−0.692	*p* > 0.05
Buttocks	0.954	*p* < 0.05	0.983	*p* < 0.05	−0.588	*p* > 0.05	−0.696	*p* > 0.05
Metacarpus	0.973	*p* < 0.05	0.976	*p* < 0.05	−0.626	*p* > 0.05	−0.638	*p* > 0.05
Metatarsus	0.976	*p* < 0.05	0.983	*p* < 0.05	−0.645	*p* > 0.05	−0.666	*p* > 0.05

**Table 6:** Serum and salivary cortisol (CORT) and lactate (LAC) concentrations in Lipizzan stallions before and after exercise (BEx and AEx) in exercise tests 1 to 4 (ExT-1 to ExT-4).

Variable [Units]	Phase of the Test	ExT-1	ExT-2	ExT-3	ExT-4
CORT serum [ng/mL]	BEx	36.4 ± 2.7 ^a^	33.4 ± 2.0	42.8 ± 4.2	35.5 ± 2.8
	AEx	50.7 ± 4.6 ^a^	35.8 ± 2.1	44.2 ± 3.8	42.0 ± 3.6
CORT saliva [ng/mL]	BEx	1.7 ± 0.2 ^b^	1.7 ± 0.2	2.1 ± 0.2	2.1 ± 0.2
	AEx	3.0 ± 0.3 ^b,A^	1.5 ± 0.2 ^A^	2.3 ± 0.3	2.3 ± 0.3
LAC [mmol/L]	BEx	0.44 ± 0.06 ^c^	0.35 ± 0.07	0.39 ± 0.08 ^c^	0.5 ± 0.1
	AEx	0.98 ± 0.2	1.0 ± 0.05	1.0 ± 0.3 ^b^	0.62 ± 0.2 ^b^

^a^ *p* < 0.001; ^b^ *p* < 0.01; ^c^ *p* < 0.05 for values with the same label in a row; ^A^ *p* < 0.001 for values with the same label in a column.

## Data Availability

The original results are available from the corresponding author.

## Abbreviations

The following abbreviations are used in this manuscript:

IRT	infrared thermography
ExT	exercise test
BEx	before the exercise
AEx	after the exercise
BST	body surface temperature
RT	rectal temperature
RR	respiratory rate
HR	heart rate
VP	blood sampling
CORT	cortisol
LAC	lactate
